# Comparative Analysis of Cytokine Expression in Oral Keratinocytes and THP-1 Macrophages in Response to the Most Prevalent Serotypes of *Aggregatibacter actinomycetemcomitans*

**DOI:** 10.3390/microorganisms9030622

**Published:** 2021-03-17

**Authors:** Daniel Betancur, Camila Muñoz Grez, Angel Oñate

**Affiliations:** Laboratory of Molecular Immunology, Department of Microbiology, Faculty of Biological Sciences, Universidad de Concepción, Concepción 4030000, Chile; dbetancur@udec.cl (D.B.); camilapmunoz@udec.cl (C.M.G.)

**Keywords:** cytokines, epithelial cells, keratinocytes, macrophages, *Aggregatibacter actinomycetemcomitans*, periodontitis

## Abstract

Background: Periodontitis is a chronic inflammatory disease associated with a dysbiotic biofilm. Many pathogens have been related with its progression and severity, one of which is *Aggregatibacter actinomycetemcomitans*, a Gram-negative bacteria with seven serotypes (a–g) according with the structure of its LPS, with serotype b defined as the most virulent compared with the other serotypes. The aim of this study was to evaluate the response of oral keratinocytes and macrophages to *A. actinomycetemcomitans*. Methods: Oral keratinocytes (OKF6/TERT2) and macrophages (THP-1) were infected with *A. actinomycetemcomitans* serotypes a, b and c. The expression of IL-1β, IL-6, IL-8, IL-18, TNF-α, MMP-9, RANKL, TLR-2, TLR-4, TLR-6, thymic stromal lymphopoietin (TSLP), and ICAM-1 was evaluated by qPCR at 2 and 24 h after infection. Results: An increase in the expression of these molecules was induced by all serotypes at both times of infection, with macrophages showing higher levels of expression at 24 h compared to epithelial cells in which the highest levels were observed in the first hours after infection. Conclusions: Keratinocytes and macrophages contribute to the inflammation in periodontitis from the early stages of infection, producing the first waves of cytokines, acting as the first signal for professional immune cell recruitment and modulation of more specific immune responses.

## 1. Introduction

According with the new classification of periodontal and peri-implant diseases and conditions, periodontitis is defined as a chronic multifactorial inflammatory disease associated with a dysbiotic biofilm, characterized by the inflammation and destruction of the tooth-supporting tissues with clinical attachment loss, alveolar bone loss, presence of periodontal pocketing and gingival bleeding [1]. This disease not only has an effect at the local level in the oral cavity, but also has an effect in systemic health, inducing low-grade chronic systemic inflammation, and being a risk factor for other conditions such as cardiovascular diseases, type 2 diabetes mellitus, premature delivery, and low birth weight, as well as being associated more recently with pathologies like Alzheimer’s disease, psoriasis, erectile dysfunction, and rheumatoid arthritis [2,3,4,5,6,7,8,9].

This pathology has an infectious etiology, associated with the presence of a pathogenic biofilm in teeth, at the subgingival level, where of the approximately 700 cultivable oral bacterial species described, 400 make up this subgingival biofilm, located in the limited space between the tooth and the gum and being the main etiological factor of periodontitis and other periodontal diseases associated with biofilms [10,11]. These bacteria, in health, are in a constant balance in terms of quantity and diversity, as well as with the host’s defenses, establishing a symbiotic state, free of inflammation that favors the growth of non-pathogenic commensal bacteria and stimulates a basal immune response [12]. However, various factors (poor oral hygiene, smoking, diabetes, individual predisposition, among others) can modify the environmental characteristics of the site and disturb the balance of this biofilm, providing the conditions for microorganisms that are normally found in low numbers, named opportunistic pathogens or pathobionts to increase in number and virulence (mainly through density-dependent cell signaling) giving place to a dysbiotic and pathogenic biofilm that favors the development and progression of this inflammatory disease [13,14,15,16].

Many pathogens have been associated with the progression and severity of this pathology, defined as *“keystone pathogens”* due to their central role in the development of the dysbiotic biofilm, and the subversion of the host’s immune system, including *Porphyromonas gingivalis* and *Aggregatibacter actinomycetemcomitans* [17,18,19].

*A. actinomycetemcomitans*, a member of the periodontal dysbiotic biofilm, is a Gram-negative, non-motile, facultative anaerobic, and capnophilic coccobacillus [20,21]. Its virulence factors can be grouped into two groups, those that are modulators of the immune system such as leukotoxin (Ltx), superantigens, cytolethal distending toxin (CDT); and a second group associated with tissue destruction which includes cell stress proteins and lipopolysaccharide (LPS) [21,22,23].

According to the structure of its LPS, seven different serotypes (a–g) have been described based on the composition of the O-polysaccharide chain of this structure, being serotypes a, b and c the most prevalent in the oral cavity in humans [24,25]. Serotype b, has been found to be the most virulent and prevalent in periodontitis patients [17], and in different cell types has shown the ability to induce a greater response in terms of production of cytokines, chemokines, cytokine receptors, and tissue destruction molecules such as metalloproteinases or receptor activator nuclear kappa ligand (RANKL), compared to serotypes a and c [26,27]. This differential response has been widely studied in cells such as dendritic cells and lymphocytes, which show a greater induction by serotype b of the production of cytokines associated with the differentiation of subpopulations of T cells Th1 and Th17, both linked to the destruction of bone tissue and chronic inflammation; however, there is little evidence about this differential character of the response induced by these serotypes in other cell types such as epithelial cells and macrophages [28,29,30].

During the pathogenesis of periodontitis, *A. actinomycetemcomitans* colonizes the gingival sulcus, infiltrating epithelial cells until reaching the underlying connective tissue, triggering an inflammatory immune response that induces the activation of mechanisms of destruction of both connective tissue and bone resorption [31,32,33]. To achieve this, *A. actinomycetemcomitans* must first break through the host’s front line of defense in the periodontium, made up of epithelial cells and macrophages. The latter, through a chemotactic gradient produced by the epithelial cells, cross the epithelium and are released into the gingival sulcus where they participate in the control of pathogens that try to colonize this site [34,35].

Extensive evidence has shown the ability of macrophages and epithelial cells to recognize periodontal pathogens, including *A. actinomycetemcomitans*, and respond to these through the production of cytokines, chemokines, antibacterial peptides, alarmins, reduction of integrins and cell–cell adhesion molecules, and others [36,37,38,39]. However, the possible differential response of these cell types against the aforementioned bacterial serotypes is not entirely clear yet, there is little knowledge about whether this variable response is a characteristic of only of professional immune cells [26,27], or if it also occurs at the first lines of host defense by modulating inflammation and tissue destruction, in a greater or lesser degree depending on the serotype, from the earliest stages of infection.

This study aimed to analyze whether the response of both oral epithelial cells and macrophages against *A. actinomycetemcomitans* presents a serotype-dependent differential character as described for other cell types [26,27,28]. For this purpose, an in vitro infection model has been developed using the most prevalent *A. actinomycetemcomitans* serotypes and oral keratinocytes and macrophages cells to simulate the initial stage of periodontitis pathogenesis.

## 2. Materials and Methods

### 2.1. Bacteria Stains

The *A. actinomycetemcomitans* strains ATCC^®^ 43717 (serotype a), ATCC^®^ 43718 (serotype b), and ATCC^®^ 43719 (serotype c) were cultured in BHI broth (70138 GranuCult. Merck^®^, Darmstadt, Germany) supplemented with 10% horse serum (H1270 Sigma-Aldrich^®^, Gillingham, UK) at 37 °C in capnophilic conditions (8% O_2_ and 12% CO_2_) for 24 h according to the growth curves previously made by our group using standard conditions. The bacteria were used at the exponential growth phase in order to obtain a reliable number of bacteria with full antigenic potential.

### 2.2. Oral Keratinocytes Culture

The immortalized human oral keratinocyte derived from floor mouth OKF6/TERT2 cell line (obtained from Dr. Rolando Vernal, Laboratory of Periodontal Biology, Universidad de Chile, Chile.) was incubated in keratinocyte serum-free medium (KSFM) (37010-022 Gibco^®^_,_ Carlsbad, CA, USA) supplemented with bovine pituitary (13028-014 Gibco^®^, Carlsbad, CA, USA), epidermal growth factor (10450-013 Gibco^®^, Carlsbad, CA, USA), calcium chloride solution 0.3M (102382 Merck^®^, Darmstadt, Germany) and penicillin/streptomycin (15140-122 Gibco^®^, Carlsbad, CA, USA) at final concentration of 100 U/mL of penicillin and 100 µg/mL of streptomycin. Keratinocytes were incubated at 37 °C in 5% CO_2_ and humidified atmosphere.

### 2.3. THP-1 Derived Macrophages Culture

The human monocytic leukemia THP-1 cell line (ATCC^®^ TIB-202) was cultured in RPMI-1640 medium (R8758 Gibco^®^, Carlsbad, CA, USA) containing 10% fetal bovine (F2442 Sigma-Aldrich^®^) serum and supplemented with glucose (G8270 Sigma-Aldrich^®^, Gillingham, UK) (14 mM final concentration), pyruvic acid (107360 Sigma-Aldrich^®^, Gillingham, UK) (1mM final concentration), 4-(2-hydroxyethyl)-1-piperazineethanesulfonic acid (HEPES) (H3375 Sigma-Aldrich^®^, Gillingham, UK) (10 mM final concentration. pH 7.35), 2-mercaptoethanol (M6250 Sigma-Aldrich^®^, Gillingham, UK) (0.5 mM final concentration) and penicillin/streptomycin (15140-122 Gibco^®^, Carlsbad, CA, USA) (100 U/mL final concentration of penicillin and 100 µg/mL of streptomycin) under a humidified atmosphere at 37 °C in 5% CO_2_.

To induce differentiation into macrophages, THP-1 cells were incubated at the same conditions in the aforementioned medium, using phorbol 12-myristate 13-acetate (PMA) (16561-29-8 Sigma-Aldrich^®^, Gillingham, UK) (100 nM final concentration) for 2 days, as previously described [40,41].

### 2.4. Infection Assay

OKF6/TERT2 monolayers were obtained by seeding 1 × 10^6^ cells per well of a 6 wells plate and incubating for 24 h at 37 °C in 5% CO_2_. In the case of THP-1 cells, these were prepared using 1 × 106 cells per well of a 6 wells plate and were activated for 48 h as mentioned above. Bacteria were grown, washed once with phosphate buffered saline solution (PBS), suspended in PBS and added to OKF6/TERT2 and THP-1 cultures at a multiplicity of infection (MOI) of 100 approximately. The plates were centrifuged at 300× *g* (RCF) for 10 min to ensure the contact between the cell layer and the bacteria; after centrifugation, plates were incubated for 90 min at 37 °C in 5% CO_2_ to allow for internalization of bacteria. Cells were then washed and incubated with fresh medium (KSFM and RPMI as appropriate) supplemented with gentamicin (G1914 Sigma-Aldrich^®^, Gillingham, UK) and metronidazole (M3761 Sigma-Aldrich^®^, Gillingham, UK), (300 µg/mL and 200 µg/mL respectively) for the post infection times defined (2 or 24 h).

### 2.5. RNA Extraction and RT-PCR

Total RNA was isolated from the cells at each condition using TRIzol^®^ Reagent (T9424 Invitrogen^®^ Sigma-Aldrich^®^, Gillingham, UK) reagent according with the manufacturer’s instructions. The reverse transcription reaction was performed using 2000 ng of the extracted RNA and using the First-Strand cDNA Synthesis SuperMix kit (18080400 Invitrogen^®^. Thermofisher^®^, Waltham, MA, USA) following the manufacturer’s protocol for reverse transcription, using DNAse digestion.

### 2.6. qPCR

The mRNA expression of cytokines and the other molecules of interest was determined by quantitative real-time polymerase chain reaction (qPCR). For this, 30 ng of cDNA were amplified using the appropriated primers (Table 1) previously designed in the platform Ensembl Genome and Primer-BLAST (NCBH-NIH), with the Takyon^®^ No Rox SYBR^®^ MasterMix dTTP Blue (UF-NSMT-B0701 Eurogentec^®^, Seraing, Belgium) reagent in an AriaMx Real-time PCR System (Agilent^®^, Santa Clara, CA, USA) as follows: 95 °C for 3 min, followed by 40 cycles of 90 °C for 5 s and 60 °C for 30 s, ending with a melt curve of 95 °C for 15 s, 60 °C for 1 min, and 95 °C for 15 s, for detection of non-specific amplification products that could lead to false positive signals. 18S rRNA expression levels were used as a normalizing endogenous control.

### 2.7. Statistical Analysis

The qPCR data were analyzed using the GraphPad Prism 8.0 software (GraphPad Software.Inc, San Diego, CA, USA) and the relative quantification was obtained by normalizing each gene mRNA expression to 18S rRNA expression using the 2^−∆∆Ct^ method [42]. The normality distribution of data was determined using Kolmogorov-Smirnov test and the differences among groups was evaluated using Tukey’s test and two ways ANOVA analysis. Asterisks were used to indicate a value of p considered statistically significant (*p* < 0.05). Data were expressed as fold-change means and standard deviation for 3 independent experiments performed at different times and qPCR reactions for each gene and sample were performed in duplicates.

## 3. Results

### 3.1. Expression of Pro-Inflammatory Cytokines and Chemokines by Oral Epithelial Cells

**Figure 1:**
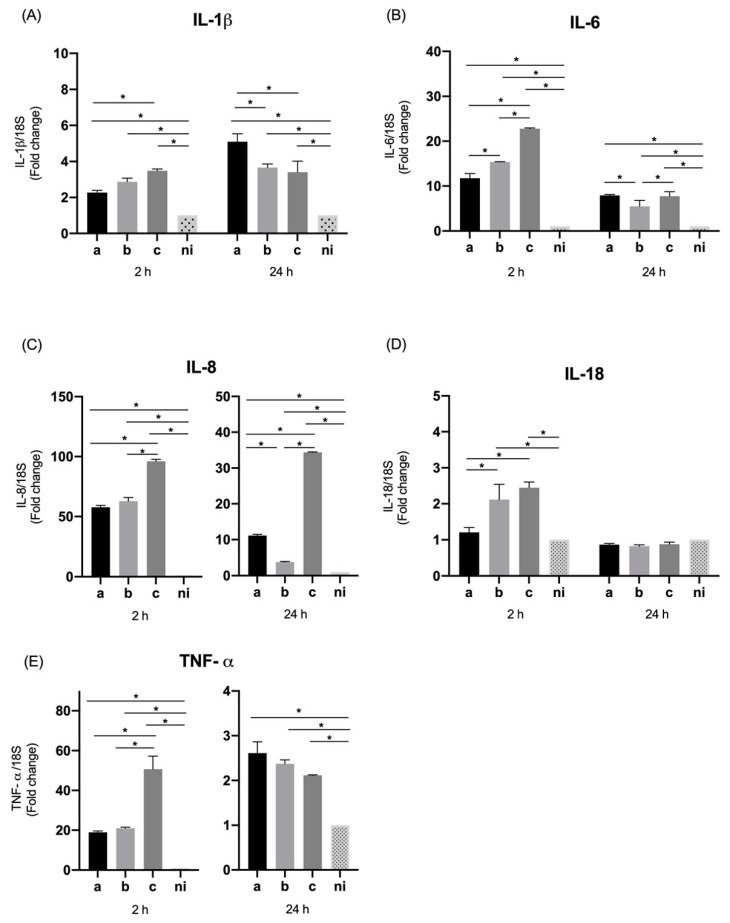
Cytokines and chemokines mRNA expression by *Aggregatibacter actinomycetemcomitans*-induced oral epithelial cells (OKF6/TERT2). Expression in oral epithelial cells infected at a MOI = 10^2^ with strains ATCC^®^ 43717^TM^ (serotype a), ATCC^®^ 43718^TM^ (serotype b), and ATCC^®^ 43719^TM^ (serotype c), 2 and 24 h after infection. For relative expression, mRNA expression in non-infected (ni) oral epithelial cells was considered as 1, as a reference for fold-change in expression using 18S rRNA expression levels as a normalizing endogenous control. Data are represented as fold-change means and standard deviation of three independent experiments performed in duplicate. (**A**) IL-1β, (**B**) IL-6, (**C**) IL-8, (**D**) IL-18, and (**E**) TNF-α. Asterisks were used to indicate a value of *p* considered statistically significant (* *p* < 0.05).

We investigated the influence of *A. actinomycetemcomitans* serotypes on expression of pro-inflammatory cytokines and chemokines by oral epithelial cells. For the case of interleukin 1 beta (IL-1β) a statistically significant overexpression was observed for the three serotypes evaluated compared to the non-infected control at 2 h (*p* = 0.0004, *p* < 0.0001, and *p* < 0.0001 for the serotypes a, b, and c respectively) as well as at 24 h, when the three serotypes are compared with the control condition (*p* < 0.0001 for all serotypes). Regarding the differences among serotypes at 2 h, the only statistically significant differences were observed between serotypes a and c, with a higher expression induced by serotype c (*p* = 0.0006). After 24 h of infection there was a statistically significant greater expression for serotype a compared with serotype b (*p* < 0.0001) and c (*p* < 0.0001), without differences between the latter two (*p* = 0.7213) (Figure 1A).

When the expression of interleukin 6 (IL-6) was measured, a statistically significant higher expression was observed for the three serotypes studied at both times of infection compared with the control condition (*p* < 0.0001); at 2 h, a statistically significant higher expression for serotype c was observed compared to the other two serotypes (*p* < 0.0001), and for serotype b over the serotype a (*p* < 0.0001). At 24 h a lower expression of this molecule was observed in all conditions compared to 2 h, although maintaining statistically significant higher relative expression for the three serotypes compared to the uninfected control, and showing statistically significant increased expression for serotypes a and c compared to serotype b (*p* = 0.0029 and *p* = 0.0055 respectively) (Figure 1B).

Interleukin 8 (IL-8 or CXCL8), showed an higher expression by epithelial cells in response to the serotypes a (*p* < 0.0001), b (*p* = 0.0007) and c (*p* < 0.0001) compared with non-infected condition at 2 h of infection. Likewise, this pattern of expression was similar at 24 h of infection for serotype a (*p* < 0.0001), b (*p* = 0.0023) and c (*p* < 0.0001) in comparison with the control condition. In terms of the differences among serotypes at 2 h, the highest expression of this molecule was induced by serotype c over serotypes a (*p* = 0.0014) and b (*p* = 0.0002), and at 24 h a statistically significant higher expression for serotype c over serotypes a and b (*p* < 0.0001) was detected, but also of serotype a over serotype b (*p* = 0.0021) (Figure 1C).

In the case of interleukin 18 (IL-18), statistically significant differences were only observed at 2 h of infection, with an overexpression of this molecule induced by serotypes b (*p* < 0.0001) and c (*p* < 0.0001) with respect to the control condition, and among serotype b (*p* < 0.0001) and c (*p* < 0.0001) over the serotype a. No statistically significant differences were observed in the expression induced by the serotype a in comparison with the non-infected condition, and no statistically significant differences were observed among conditions at 24 h (Figure 1D).

On the other hand, when the levels of tumor necrosis factor alpha (TNF-α) were measured, a statistically significant increase in expression was observed by the cells studied at 2 h of stimulation in response to the serotype a (*p* < 0.0001), b (*p* < 0.0001) and c (*p* = 0.0148) in comparison with the non-infected condition; in the same way, this over expression was observed also at 24 h with statistically significant differences for the serotype a (*p* = 0.0199), b (*p* = 0.0030), and c (*p* < 0.0001) compared with the non-infected condition. Regarding the differences among serotypes, statistically significant higher levels of TNF-α were expressed only after 2 h of infection by serotype c compared to serotypes a (*p* = 0.0354) and b (*p* = 0.0440) (Figure 1E).

### 3.2. Expression of Molecules Associated with Tissue Destruction by Oral Epithelial Cells

**Figure 2:**
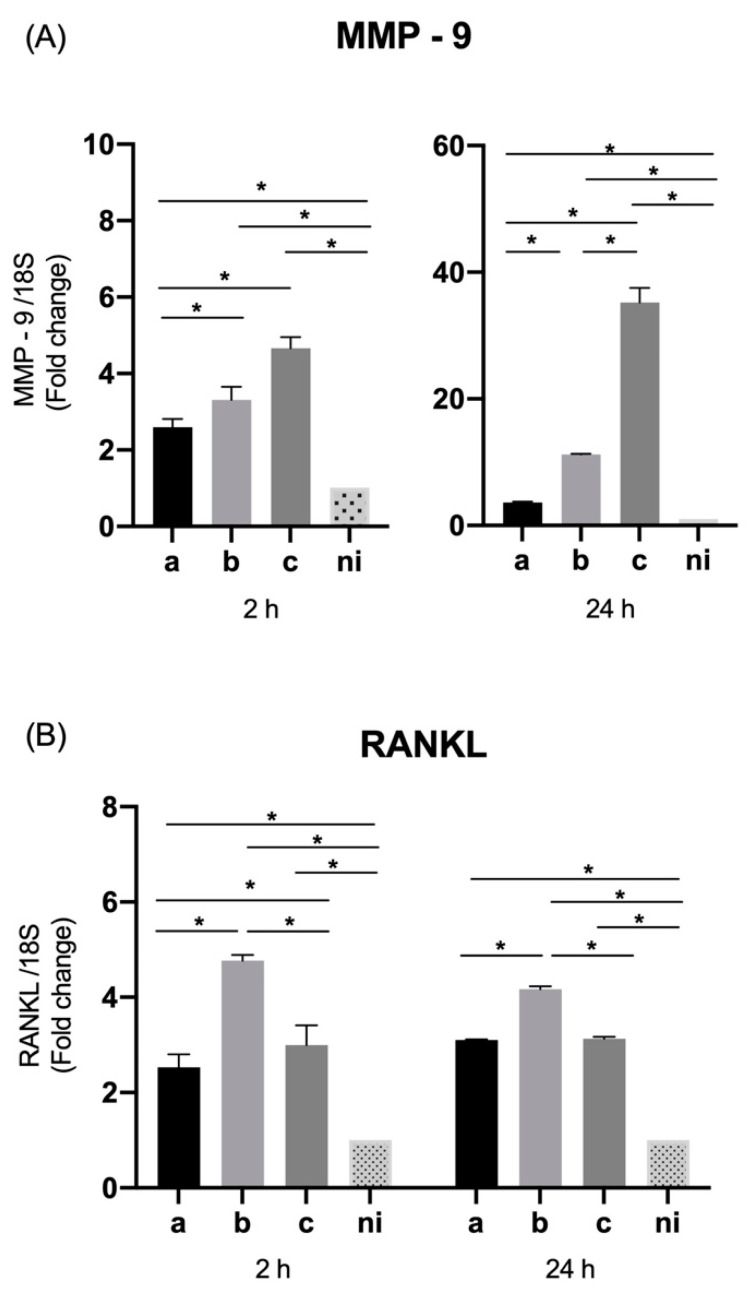
Molecules associated with tissue destruction mRNA expression by *A. actinomycetemcomitans*-induced oral epithelial cells (OKF6/TERT2). Expression in oral epithelial cells infected at a MOI = 10^2^ with strains ATCC^®^ 43717^TM^ (serotype a), ATCC^®^ 43718^TM^ (serotype b), and ATCC^®^ 43719^TM^ (serotype c), 2 and 24 h after infection. For relative expression, mRNA expression in non-infected (ni) oral epithelial cells was considered as 1, as a reference for fold-change in expression using 18S rRNA expression levels as a normalizing endogenous control. Data are represented as fold-change means and standard deviation of three independent experiments performed in duplicate. (**A**) MMP-9. (**B**) RANKL. Asterisks were used to indicate a value of *p* considered statistically significant (* *p* < 0.05).

When the levels of metalloproteinase 9 (MMP-9) were measured, a statistically significant increased expression was observed in response to the serotype a (*p* = 0.0158), b (*p* = 0.0187), and c (*p* = 0.0052) with respect to non-infected condition at 2 h of stimulation, and in response to serotype a (*p* = 0.0002), b (*p* < 0.0001) and c (*p* = 0.0033) compared with the control condition after 24 h of infection. Regarding the differences among serotypes, serotype c was able to induce statistically significant higher expression levels of this molecule than serotype a (*p* = 0.0438), and the same way serotype b over serotype a (*p* = 0.0430), at 2 h of infection in both cases. On the other hand, when the differences among serotypes are analyzed after 24 h of stimulation statistically significant higher relative expression of serotype c over the serotypes a (*p* = 0.0046) and b (*p* = 0.0083) becomes evident, as well as of the serotype b over the serotype a (*p* < 0.0001) (Figure 2A).

Regarding expression levels of RANKL, the serotypes a (*p* < 0.0001), b (*p* < 0.0001) and c (*p* < 0.0001) were capable of inducing a statistically significant overexpression of this molecule compared to the non-infected condition at both times of infection. At two hours we observed higher levels of RANKL expression for serotype b over serotypes a (*p* < 0.0001) and c (*p* < 0.0001) and the serotype c over the serotype a (*p* = 0.0287). On the other hand, at 24 h of stimulation statistically significant differences in the expression were observed regarding the b serotype in comparison with the serotype a (*p* < 0.0001) and c (*p* < 0.0001) (Figure 2B).

### 3.3. Expression of TLR Receptors in Oral Epithelial Cells

**Figure 3:**
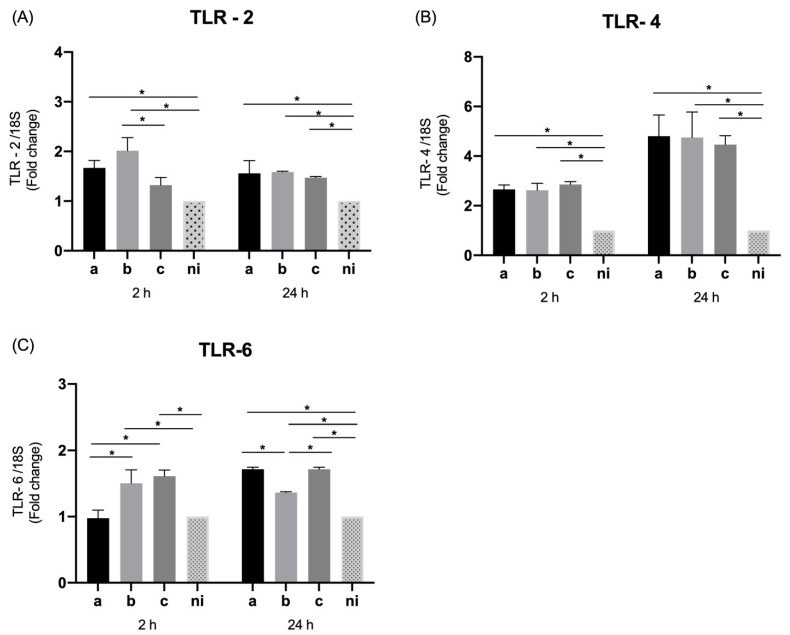
Toll like receptors (TLR) mRNA expression by *A. actinomycetemcomitans*-induced oral epithelial cells (OKF6/TERT2). Expression in oral epithelial cells infected at a MOI = 10^2^ with strains ATCC^®^ 43717^TM^ (serotype a), ATCC^®^ 43718^TM^ (serotype b), and ATCC^®^ 43719^TM^ (serotype c), 2 and 24 h after infection. For relative expression, mRNA expression in non-infected (ni) oral epithelial cells was considered as 1, as a reference for fold-change in expression using 18S rRNA expression levels as a normalizing endogenous control. Data are represented as fold-change means and standard deviation of three independent experiments performed in duplicate. (**A**) TLR-2, (**B**) TLR-4, and (**C**) TLR-6. Asterisks were used to indicate a value of *p* considered statistically significant (* *p* < 0.05).

In the case of TLR-2, at 2 h post infection, a statistically significant increase in its expression was observed in response to serotypes a (*p* = 0.0003) and b (*p* < 0.0001) compared to the uninfected condition, without statistically significant difference between the serotype c and control condition (*p* = 0.0821). When comparing serotypes, the expression of this molecule was statistically significant higher for serotype b than for c (*p* = 0.0002), with no statistically significant differences among serotype a and the remaining serotypes. On the other hand, at 24 h of infection, a statistically significant overexpression was only observed among the infected conditions and the control condition (*p* = 0.0018, *p* = 0.0011, and *p* = 0.0073 for the serotypes a, b, and c respectively), without statistically significant differences among serotypes (Figure 3A).

In terms of the expression of TLR-4, at 2 h of infection, a statistically significant overexpression was observed induced by the serotype a (*p* = 0.0048), b (*p* = 0.0055) and c (*p* = 0.0018) compared to the control condition; and likewise at 24 h of stimulation a higher levels of transcription were detected in response to the three serotypes over the non-infected condition (*p* < 0.0001); no evidence of statistically significant differences among serotypes was observed for 2 h and 24 h after infection (Figure 3B).

When levels of TLR-6, are measured, a statistically significant higher expression can be observed after 2 h of infection when the cells are stimulated with serotypes b (*p* < 0.0001) and c (*p* < 0.0001) compared to the uninfected condition, with no differences between serotype a and the control condition (*p* = 0.9880). While respecting the differences in expression among serotypes, a statistically significant higher expression was observed for serotypes b (*p* < 0.0001) and c (*p* < 0.0001) over serotype a. When TLR-6 expression is observed after 24 h of infection, a statistically significant overexpression of this molecule is evidenced for all stimulated conditions, for serotypes a (*p* < 0.0001), b (*p* = 0.0009), and c (*p* < 0.0001) compared to the non-infected control. While regarding the differences among infected conditions, the lowest expression is given by serotype b, being statistically significant higher for serotypes a (*p* = 0.0013) and c (*p* = 0.0013) over serotype b, without differences between them (Figure 3C).

### 3.4. Expression of Thymic Stromal Lymphopoietin (TSLP) and ICAM-1 by Oral Epithelial Cells

**Figure 4:**
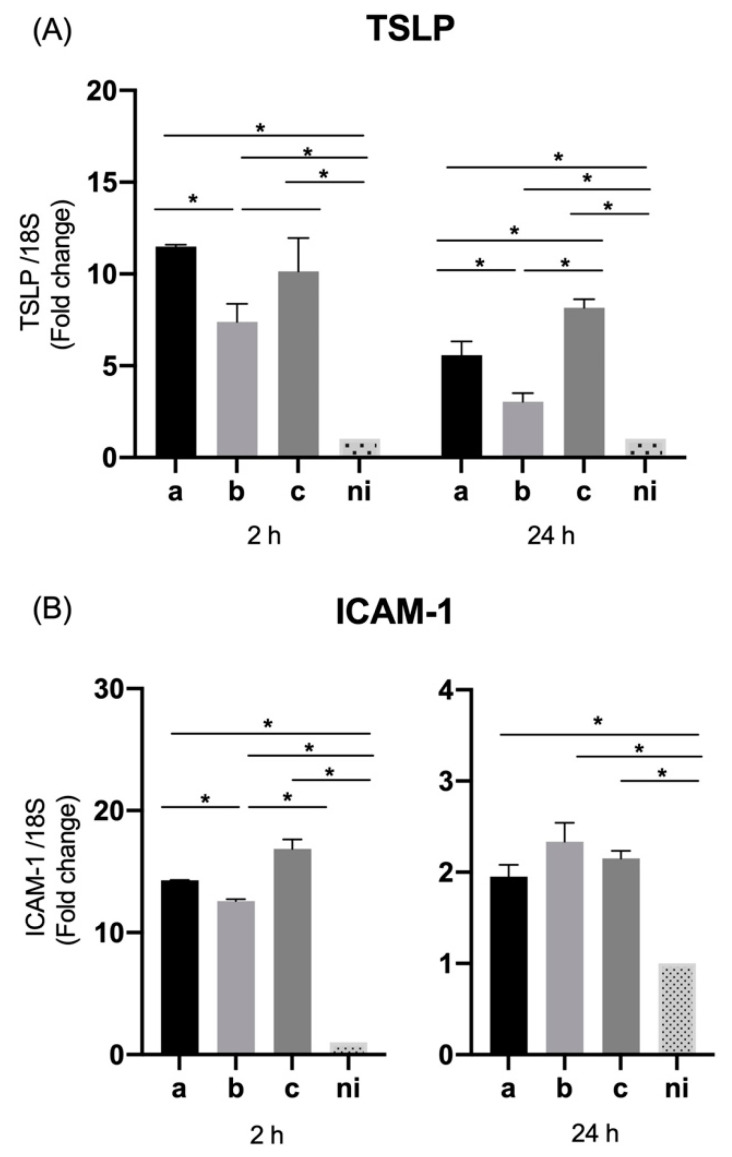
Thymic stromal lymphopoietin (TSLP) and ICAM-1 mRNA expression by *A. actinomycetemcomitans*-induced oral epithelial cells (OKF6/TERT2). Expression in oral epithelial cells infected at a MOI = 10^2^ with strains ATCC^®^ 43717^TM^ (serotype a), ATCC^®^ 43718^TM^ (serotype b), and ATCC^®^ 43719^TM^ (serotype c), 2 and 24 h after infection. For relative expression, mRNA expression in non-infected (ni) oral epithelial cells was considered as 1, as a reference for fold-change in expression using 18S rRNA expression levels as a normalizing endogenous control. Data are represented as fold-change means and standard deviation of three independent experiments performed in duplicate. (**A**) TSLP. (**B**) ICAM-1. Asterisks were used to indicate a value of *p* considered statistically significant (* *p* < 0.05).

In the case of TSLP levels of expression, the serotypes a (*p* < 0.0001), b (*p* < 0.0001), and c (*p* < 0.0001), induced an overexpression of this molecule compared to the non-infected condition at 2 h of infection; similarly, at 24 h of infection, serotype a (*p* < 0.0001), b (*p* = 0.0327), and c (*p* < 0.0001) were able to induce statistically significant higher relative expression than the uninfected condition. Regarding the differences among serotypes, the expression was statistically significant higher for the serotype a (*p* < 0.0001) and c (*p* = 0.0037) over serotype b at 2 h; and for the serotype a (*p* = 0.0076) and c (*p* < 0.0001) over the serotype b, and for the serotype c over the serotype a (*p* = 0.0064) at 24 h after infection (Figure 4A).

The expression of ICAM-1 in the studied conditions was statistically significant higher for the serotype a (*p* < 0.0001), b (*p* < 0.0001), and c (*p* = 0.0006) respect to the control condition at 2 h and for the serotype a (*p* = 0.0152), b (*p* = 0.0201), and c (*p* = 0.0039) compared with control condition at 24 h after infection. In relation to the differences among serotypes, only at 2 h of infection variations were observed with statistically significant higher levels of expression induced by the serotype a (*p* = 0.0035) and c (*p* = 0.0035) over serotype b (Figure 4B).

### 3.5. Expression of Pro-Inflammatory Cytokines and Chemokines by THP-1 Macrophage Cells

**Figure 5:**
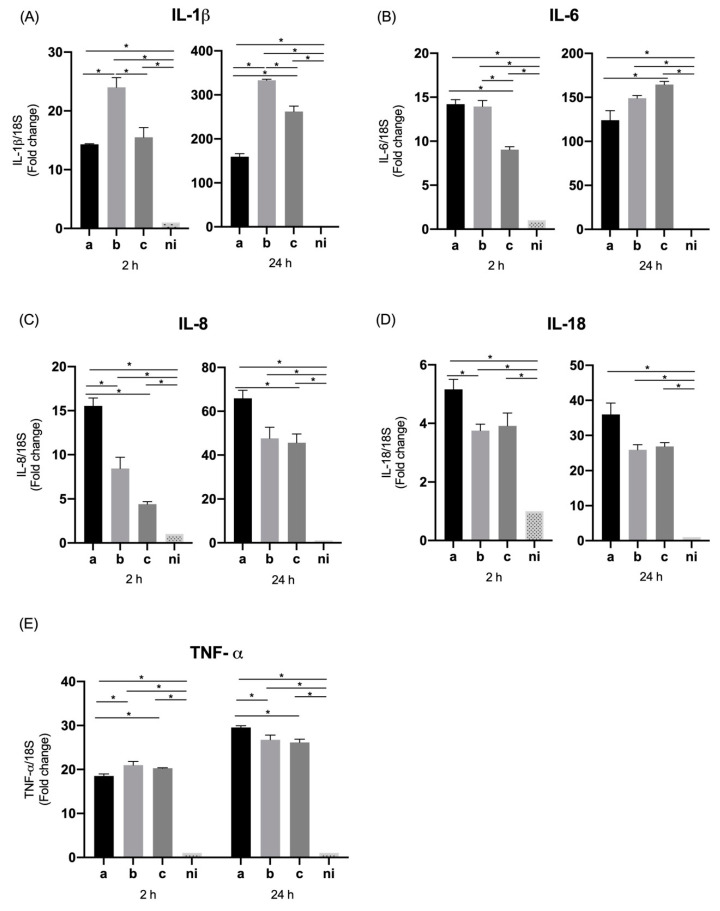
Cytokines and chemokines mRNA expression by *A. actinomycetemcomitans*-induced THP-1 human macrophages (ATCC^®^ TIB-202™). Expression in macrophages infected at a MOI = 10^2^ with strains ATCC^®^ 43717^TM^ (serotype a), ATCC^®^ 43718^TM^ (serotype b), and ATCC^®^ 43719^TM^ (serotype c), 2 and 24 h after infection. For relative expression, mRNA expression in non-infected (ni) macrophages was considered as 1, as a reference for fold-change in expression using 18S rRNA expression levels as a normalizing endogenous control. Data are represented as fold-change means and standard deviation of three independent experiments performed in duplicate. (**A**) IL-1β, (**B**) IL-6, (**C**) IL-8, (**D**) IL-18, and (**E**) TNF-α. Asterisks were used to indicate a value of *p* considered statistically significant (* *p* < 0.05).

The qPCR data reveal that in the case of IL-1β, the serotypes a (*p* < 0.0001), b (*p* = 0.0041) and c (*p* = 0.0108) induced a statistically significant overexpression with respect to the non-infected condition at two hours; similarly, at 24 h of infection by the serotype a (*p* = 0.0003), b (*p* < 0.0001) and c (*p* = 0.005) respect to the non-infected control. When the differences among serotypes are analyzed at 2 h, a statistically significant greater expression is induced by serotype b over serotypes a (*p* = 0.0228) and c (*p* = 0.0310), and in the case of 24 h post infection, the highest expression is given by serotype b on serotypes a (*p* = 0.0006) and c (*p* = 0.03), followed by serotype c on serotype a (*p* = 0.0226) (Figure 5A).

In the case of IL-6, at 2 h post infection all the serotypes studied induce a statistically significant greater expression of this molecule compared to the uninfected condition, serotype a (*p* < 0.0001), b (*p* = 0.0010) and c (*p* = 0.0002), being also statistically significant higher for serotypes a (*p* = 0.0064) and b (*p* < 0.0275) than for serotype c. Similarly, at 24 h, all infected conditions showed statistically significant higher expression levels of IL-6 than the uninfected condition, serotype a (*p* = 0.0066), b (*p* < 0.0001), and c (*p* < 0.0001), showing statistically significant differences only between serotype a and serotype c (*p* = 0.0315) (Figure 5B).

The IL-8 expression data by macrophage cells samples showed a statistically-significant increased expression of this cytokine in response to the three serotypes analyzed compared to the unstimulated control condition, serotype a (*p* = 0.0025), b (*p* = 0.0231), and c (*p* = 0.0062) at 2 h of infection and serotype a (*p* = 0.0014), b (*p* = 0.0102) and c (*p* = 0.0070) at 24 h of infection. On the other hand, the differences among bacteria revealed that at 2 h the highest expression was induced by serotype a over serotype b (*p* = 0.0006) and c (*p* = 0.0099), without statistically significant differences between the latter two. While at 24 the overexpression induced by serotype a alone was statistically significant higher compared to serotype c (*p* = 0.0292), without statistically significant differences with respect to serotype b (*p* = 0.1630), or between serotypes b and c (*p* = 0.9675) (Figure 5C).

In terms of the expression of IL-18 by THP-1 macrophage cells, the three serotypes analyzed were able to induce a statistically significant expression of this cytokine with respect to the control condition serotype a (*p* = 0.0056), b (*p* = 0.0051) and c (*p* = 0.0190) at 2 h of infection and serotype a (*p* = 0.0074), b (*p* = 0.0017) and c (*p* = 0.0002) at 24 h of infection. There were no statistically significant differences among serotypes, except for a significantly greater expression of serotype a over serotype b (*p* = 0.0066) at 2 h of infection (Figure 5D).

We also evaluated the expression of TNF- α by macrophage cells infected with the 3 previously mentioned serotypes of *A. actinomycetemcomitans*, observing for both time points studied and all serotypes, the ability to induce a statistically significant overexpression of this cytokine compared to the uninfected condition (*p* < 0.0001). The analysis of the differences among serotypes showed at 2 h significantly higher levels of expression in response to serotypes b (*p* = 0.0005) and c (*p* < 0.0098) over serotype a, without marked differences between serotype b and c; while on the contrary, at 24 h, the most considerable overexpression was given by serotype a over serotypes b (*p* < 0.0001) and c (*p* < 0.0001), without statistically significant differences between the latter two (Figure 5E).

### 3.6. Expression of MMP-9 and RANKL by THP-1 Macrophage Cells

As evaluated for epithelial cells, the expression MMP-9 was measured in THP-1 cells in response to the aforementioned bacterial serotypes.

**Figure 6:**
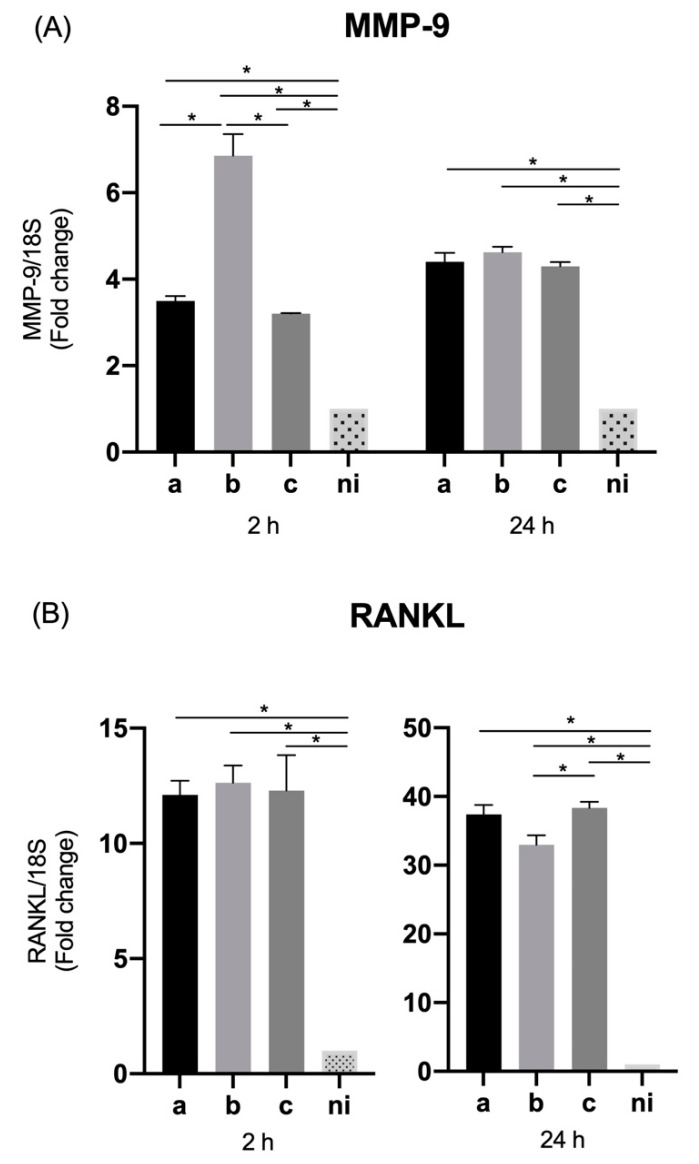
Molecules associated with tissue destruction mRNA expression by *A. actinomycetemcomitans*-induced THP-1 human macrophages (ATCC^®^ TIB-202™). Expression in macrophage cells infected at a MOI = 10^2^ with strains ATCC^®^ 43717^TM^ (serotype a), ATCC^®^ 43718^TM^ (serotype b), and ATCC^®^ 43719^TM^ (serotype c), 2 and 24 h after infection. For relative expression, mRNA expression in non-infected (ni) macrophages was considered as 1, as a reference for fold-change in expression using 18S rRNA expression levels as a normalizing endogenous control. Data are represented as fold-change means and standard deviation of three independent experiments performed in duplicate. (**A**) MMP-9. (**B**) RANKL. Asterisks were used to indicate a value of *p* considered statistically significant (* *p* < 0.05).

MMP-9 showed a significantly increased expression (*p* < 0.0001) in response to stimulation with the three serotypes used with respect to the control condition at both times analyzed. Showing statistically significant differences among serotypes only at 2 h, with a higher expression of serotype b over serotypes a (*p* < 0.0001) and c (*p* < 0.0001), with no marked difference between the latter, while at 24 h no differences in expression were observed among serotypes (Figure 6A).

For its part, RANKL was significantly overexpressed in response to the 3 bacteria studied compared to the non-infected condition, serotype a (*p* = 0.0015), b (*p* = 0.0028), and c (*p* = 0.0154) at 2 h of infection and serotype a (*p* < 0.0001), b (*p* = 0.0002), and c (*p* < 0.0001) at 24 h of infection. The differences among serotypes were observed only 24 h after infection with a significantly greater overexpression of serotype c over serotype b (*p* = 0.0073), and without statistically significant differences with serotype a (*p* = 0.8094) or differences between serotypes a and b (*p* = 0.1856) (Figure 6B).

### 3.7. Expression of TLR Receptors in THP-1 Macrophage Cells

**Figure 7:**
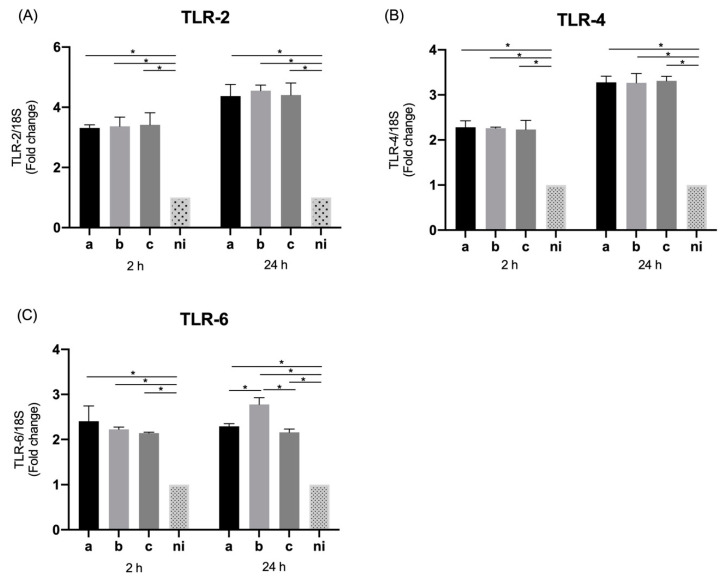
Toll like receptors (TLR) mRNA expression by *A. actinomycetemcomitans*-induced THP-1 human macrophages (ATCC^®^ TIB-202™). Expression in macrophages infected at a MOI = 10^2^ with strains ATCC^®^ 43717^TM^ (serotype a), ATCC^®^ 43718^TM^ (serotype b), and ATCC^®^ 43719^TM^ (serotype c), 2 and 24 h after infection. For relative expression, mRNA expression in non-infected (ni) macrophages was considered as 1, as a reference for fold-change in expression using 18S rRNA expression levels as a normalizing endogenous control. Data are represented as fold-change means and standard deviation of three independent experiments performed in duplicate. (**A**) TLR-2, (**B**) TLR-4, and (**C**) TLR-6. Asterisks were used to indicate a value of *p* considered statistically significant (* *p* < 0.05).

We also evaluate the expression of TLR-2, TLR-4, and TLR-6 by THP-1 cells in response to infection with the aforementioned bacterial serotypes. The qPCR data reveal that the expression of these three receptors was significantly higher (*p* < 0.0001) in response to the three serotypes studied both at 2 and 24 h post infection (Figure 7), with no differences among serotypes for any of the aforementioned receptors at both times studied, with exception of a significantly higher overexpression of TLR-6 at 24 h induced by serotype b over serotypes a (*p* = 0.0027) and c (*p* = 0.0002) without marked differences between the latter two (*p* = 0.6301) (Figure 7C).

### 3.8. Expression of TSLP and ICAM-1 in THP-1 Macrophage Cells

Similar to what was done in epithelial cells, the expression levels of TSLP and ICAM-1 were measured in THP-1 cells infected with the three bacterial serotypes under study.

**Figure 8:**
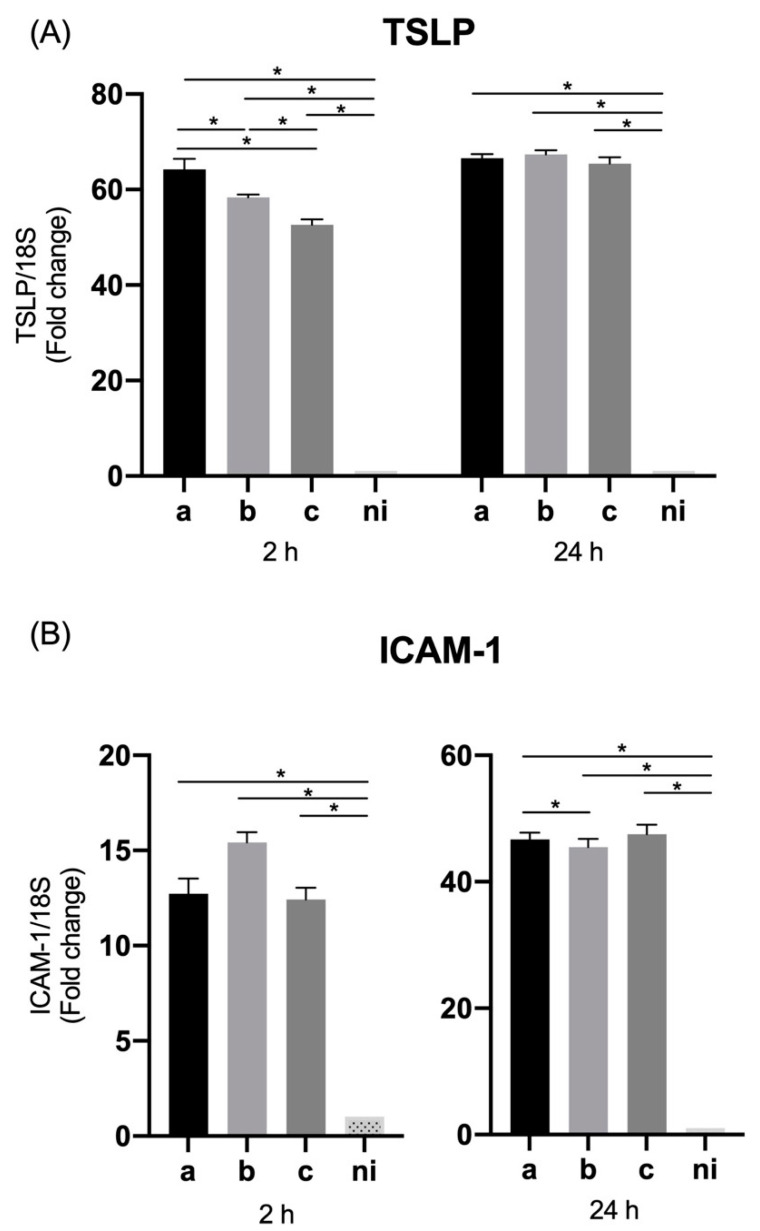
TSLP and ICAM-1 mRNA expression by *A. actinomycetemcomitans*-induced THP-1 human macrophages (ATCC^®^ TIB-202™). Expression in macrophages infected at a MOI = 10^2^ with strains ATCC^®^ 43717^TM^ (serotype a), ATCC^®^ 43718^TM^ (serotype b), and ATCC^®^ 43719^TM^ (serotype c), 2 and 24 h after infection. For relative expression, mRNA expression in non-infected (ni) macrophages was considered as 1, as a reference for fold-change in expression using 18S rRNA expression levels as a normalizing endogenous control. Data are represented as fold-change means and standard deviation of three independent experiments performed in duplicate. (**A**) TSLP, (**B**) ICAM-1. Asterisks were used to indicate a value of *p* considered statistically significant (* *p* < 0.05).

TSLP showed significantly higher levels of expression (*p* < 0.0001) by THP-1 cells for the three serotypes used, compared to the uninfected condition at both 2 and 24 h. While the differences among serotypes were only statistically significant at 2 h, showing a greater expression in response to serotype a over serotypes b (*p* < 0.0001) and c (*p* < 0.0001), and serotype b over serotype c (*p* < 0.0001). No differences were observed among serotypes at 24 h of infection (Figure 8A).

Regarding the expression levels of ICAM-1 in response to infection with *A. actinomycetemcomitans*, all the serotypes studied were capable of inducing a statistically significant increase in the expression of this molecule compared to the non-stimulated condition, serotype a (*p* = 0.0035), b (*p* < 0.00201), and c (*p* = 0.0011) at 2 h of infection, and serotype a (*p* < 0.0001), b (*p* < 0.0001), and c (*p* < 0.0001) at 24 h of infection (Figure 8B).

No marked differences were observed among serotypes, except at 24 h between serotypes a and b (*p* = 0.0291), being greater for serotype a versus serotype b (Figure 8B).

The fold changes of mRNA relative expression for all molecules mentioned above are summarized in the Table 2.

## 4. Discussion

Epithelial cells and macrophages are a natural physical barrier and the first defense mechanisms at the level of periodontal sulcus or pocket, preventing the penetration of microorganisms into the deep connective tissue. They also act as a surveillance system to mount an inflammatory response to microbial colonization, expression of cytokines, chemokines, tissue destruction molecules, and modification of the expression of receptors associated with the recognition of microorganisms [43].

Regarding the expression of cytokines, our data are consistent with the available evidence that supports the expression of inflammatory mediators by macrophages and epithelial cells in response to periodontal pathogens, showing an increase in these molecules at the level of the gingival crevicular fluid in sites that exhibit destruction of periodontal tissues [44,45,46,47].

The role of cytokines in the progression of periodontitis is extremely important. These molecules are modulators of both homeostasis and inflammation, being the first response signal against pathogens by stimulating the barrier site and communicating it with professional immune cells such as T and B lymphocytes, dendritic cells, and NK cells [48]. The cytokines participating in the immune response against periodontal pathogens can be categorized into three stages. A first wave of cytokines expressed by epithelial cells, gingival fibroblasts and innate immune cells, produced directly due to the contact of the microorganisms with the host cells. A second group of cytokines associated with the differentiation of specific subpopulations of lymphocytes, secreted by polymorphonuclear cells, antigen-presenting cells, phagocytes and local lymphocytes in response to the stimulus of the microbiome. Finally, a third wave of cytokines, expressed by the already differentiated lymphocyte subpopulations, which participates either as feedback mechanisms or direct effector of inflammation and activation of tissue destruction pathways in case of disease [49].

IL-1β, a cytokine member of the IL-1 family involved in both innate and adaptive immunity and associated with inflammation, is overexpressed by both epithelial and macrophage cells in response to the three bacterial serotypes studied, as shown by Dickinson et al., where gingival epithelial cells were stimulated with *A. actinomycetemcomitans* VT1169 at 2 and 24 h, showing a greater secretion of IL-1β, TNF-α, IL-8, and IL-6 mainly at 24 h compared to the control condition [50]. There is little evidence that analyzes the expression of IL-1β in macrophages comparing among *A. actinomycetemcomitans* serotypes, however in a study in which macrophage-like cells were pretreated for 24 h with *A. actinomycetemcomitans* LPS, high concentrations of IL-1β were observed in the cell supernatant compared to the control, this study also highlights that the LPS of *A. actinomycetemcomitans* induces a tolerance response in macrophages that alters the secretion of IL- β and TNF- α as well as of the tissue-degrading enzyme MMP-9, modulating the host inflammatory response [51].

In the case of IL-18, a chemokine whose main activity is polynuclear neutrophil attractant with an important role in periodontitis development and described as critical in driving the pathologic breakdown of barrier integrity in different cells models, our results are consistent with those obtained in other studies such as Ando-Suguimoto et al., where the transcription of IL -1β and IL-18 was upregulated in macrophages and oral epithelial cells after 1 h interaction with *A. actinomycetemcomitans*, and similar to our results, this positive regulation persisted only in macrophages, in our case for 24 h post infection [52].

There is little evidence of the role of IL-18, a pro-inflammatory cytokine also known as an interferon-gamma inducing factor in the response to periodontal pathogens, however the evidence available in other epithelial cell models suggests this molecule plays a key function in the control of the barrier function of epithelia, being critical in driving the pathologic breakdown of barrier integrity allowing the passage of microorganisms to sub-epithelial tissues [53,54], and probably its dissemination regulating the expression of matrix metalloproteinase 9 (MMP-9) [55].

Both IL-1β and IL-18 expression have been described as a consequence of the activation of the inflammasome, a macromolecular complex that, through various signals, participates in the processing of inflammatory pro-interleukins to their active forms. These signals range from the recognition of molecular patterns associated with microorganisms (MAMPs) and damage (DAMPs), as well as the entry of various molecules, and in the case of the entry of some virulence factors into cells [56,57].

The expression of NLRP3, which is involved in inflammasomes, plays a key role in periodontitis, being higher in periodontitis gingival tissues than in healthy tissues, especially at the epithelium layer [58]. And at the salivary level, higher levels of NLRP3 were found in cases of periodontitis compared to healthy subjects [59].

Some components of the inflammasome, such as apoptosis-associated speck-like protein containing a CARD (ASC) and absent in melanoma 2 (AIM2) proteins and NLRP3, are upregulated by *A. actinomycetemcomitans* infection in THP-1 cells and human mononuclear leukocytes [60,61]. However, in the case of epithelial cells, different signaling and activation pathways of the inflammasome are induced in the response against *A. actinomycetemcomitans*, where it has been shown that this bacterium enhances the expression of NLRP3, TLR4, TLR2, and NOD2 in macrophages but not in human gingival epithelial cells [52].

In our case, the differences in magnitude in the expression of IL-1β and IL-18 between macrophages and oral epithelial cells, could reinforce the idea that the response to a pathogen not only depends on the stimulus (type of bacteria, serotype, strain, etc.) but also on cell type, as in the case of osteoblastic cells which, when stimulated with *A. actinomycetemcomitans*, apoptosis is promoted at least partially through the NLRP3 inflammasome activation [62].

The future study of the expression of inflammasome components in response to the different serotypes of *A. actinomycetemcomitans* and the role of its virulence factors, could be a way to elucidate the mechanisms of the differential response against this bacterium that is observed only in some cell types such as professional immune cells, but not in innate immune cells [26,27,28,29,30].

MMP-9, the main gelatinase in oral fluids involved in the degradation of the extracellular matrix [63], is expressed by various cell types and activated or released by pro-inflammatory cytokines such as IL-1 β, TNF- α and some proteases derived from pathogens of the periodontal biofilm [64]. Our results for the cells types studied, suggests that it begins to be expressed by epithelial cells from the initial stages of infection of periodontal tissues, similar to how it occurs in other epithelia and macrophages [65,66,67]. In the case of macrophages, the LPS of *A. Actinomycetemcomitans* induces the expression of MMP-9 as described previously [68]; likewise, in our hands, using the complete bacteria, we found a higher expression of this molecule but without a differential expression pattern by a specific serotype, except at 2 h on the part of serotype b compared with serotypes a and c, but reaching similar levels for all serotypes at 24 h post-infection.

The expression of RANKL, a molecule associated with bone destruction for its ability to activate osteoclasts, by oral epithelial cells and macrophages has been extensively studied, with special attention to the effect of other cytokines such as TNF- α on the modulation of its expression, particularly at the epithelial level [69,70,71]. There is little evidence regarding the effect of the stimulation of oral epithelial cells with periodontal microorganisms and its effect on the expression of this mediator of bone destruction. As such, the expression levels by epithelial cells and macrophages stimulated with the three *A. actinomycetemcomitans* serotypes suggest that these cell types support bone destruction, from the earliest stages of infection, or at least configure an inflammatory environment conducive to other cells, such as osteoclasts or pre-osteoclasts to activate their bone destruction mechanisms.

*A. actinomycetemcomitans* has lipopolysaccharide, an endotoxin recognized by TLR2 (a molecule that recognizes acylated bacterial lipoproteins and signals as a heterodimer with either TLR1 or TLR6) and TLR4 (a transmembrane protein that participates in the recognition of LPS in Gram-negative bacteria), being TLR4 the main receptor for LPS [72]. The overexpression of these receptors together with their role in the expression of pro-inflammatory cytokines by various cell types, in response to periodontal pathogens has been demonstrated in vitro and in vivo; for example, macrophages TLR2 -/- and TLR4 -/- stimulated with *A. actinomycetemcomitans* exhibited an attenuated production of the proinflammatory cytokines TNF-α and IL-6 [72].

On the other hand, it has been observed that the expression of TLR2 and TLR4 in gingival epithelial cells is higher in individuals with diabetes and is positively regulated according to the severity of periodontal disease [73], and that TLR2 and TLR4 would have a role in the control of infection by *A. actinomycetemcomitans* [74,75]. This evidence is consistent with our results that show an increase in the expression of toll-like receptors on both macrophages and epithelial cells in response to the three bacterial serotypes at both time points analyzed. However, in the case of TLR6, even though its expression has been demonstrated both in oral epithelial cells and in macrophages, there are no studies to date that analyze in depth its role in the cellular response against bacteria with structural variables such as those of *A. actinomycetemcomitans*, both independently or as forming TLR2/TLR6 dimers. In our case, and for the previously described experimental conditions, a discrete increase in TLR6 expression could be observed regardless of the serotype used, mainly in macrophage cells. However, these data could be different under other experimental conditions or reference parameters.

The expression and function of TLR in the oral cavity are key to the maintenance of homeostasis of oral tissues, given the constant presence of commensal microorganisms. The expression of these receptors is not only mediated by the state of the tissues (inflamed versus non-inflamed) but they are also regulated in terms of their location. TLR2 and TL4 are highly expressed in the epithelial basal layers but less so in the cells of the superficial layers where the exposure to the environment and microorganisms is greater [76], an aspect against which the analysis of this study is limited, as we worked with a cell monolayer models.

More studies on the role of Toll-like receptors in the recognition of the structural variables of *A. actinomycetemcomitans* are required, mainly for the case of TLR4. This receptor dimerized with myeloid differentiation factor 2 (MD-2) forms the heterodimer responsible for recognizing a common pattern in the structurally diverse LPS molecules [77]. Lipid A corresponds to the conserved molecular pattern of LPS and is the main inducer of the immune response against LPS, with TLR4 together with MD-2 being responsible for the physiological recognition of LPS [78]. In this sense, additional evidence is required to support how this receptor capable of recognizing the conserved fraction of LPS (lipid A), could be modulating a serotype-dependent differential immune response (as it occurs in some cell types) [26,27,28,29,30], based on structural variables typical of the O antigen, a region not described as recognized by this receptor. Probably other receptors such as NOD2 or NLRP3, and other virulence factors could be contributing to the inflammatory response induced by these bacteria [79].

Thymic stromal lymphopoietin (TSLP) an analogous of IL-7 cytokine, is a critical factor linking responses at interfaces between the body and the environment, expressed by various cell types such as epithelial cells and epidermal keratinocytes, mast cells, airway smooth muscle cells, fibroblasts, dendritic cells, trophoblasts, and cancer or cancer-associated cells [80]. Environmental factors such as Toll-like receptor ligands, a NOD2 ligand, viruses, microbes, allergen sources and cigarette smoke trigger TSLP production, and proinflammatory cytokines induce or enhance TSLP production [80,81]. To date, there is no evidence of the expression of this molecule by macrophages and oral epithelial cells in response to periodontal pathogens such as *A. actinomycetemcomitans.* However, our data show that *A. actinomycetemcomitans* has the ability to induce TSLP expression in epithelial cells and to a much greater extent in macrophages compared to unstimulated cells, opening the possibility of studying this molecule as one of the key mediators in the response of the first cells that come into contact with the pathogen in the initial stages of periodontitis.

In the case of ICAM-1, also known as CD54, an increase in its expression has been described in macrophages stimulated with *A. actinomycetemcomitans* LPS [82], as well as gingival epithelial cells induced with 5 different strains of *A. actinomycetemcomitans* [83,84]. Our data are in agreement with the available evidence showing an increase in ICAM-1 expression for both types of cells studied in response to the three bacterial serotypes, without a sustained tendency to a differential response of one serotype over another. Based on these data, the role of periodontal pathogens in the increase of intercellular adhesion molecules can be affirmed, which could serve as a mechanism to retain inflammatory cells at the site of infection, limiting the spread of pathogens and concentrating inflammatory cells from the first contact by the host with the microorganisms [85,86].

The molecules expressed by the cell types used in this study correspond to molecules belonging to the first and second wave of mediators expressed in the initial stages of periodontitis. In the case of epithelial cells, for most of the molecules studied, the highest levels of expression were observed at 2 h post stimulation, probably due to the ability of these cells to be rapid responders and to give the first warning signal when encountering some pathogens, contrary to what happened in macrophages, where a large part of the genes evaluated showed their highest expression levels at 24 h post-infection. In this way, the synchrony as a function of time of the different cell types present in periodontal tissues is confirmed, from keratinocytes and macrophages involved in the first discharges of inflammatory mediators to the subsequent expression of molecules by specialized cell subpopulations capable of inducing specific cytokines profiles. [27,28]

In agreement with this, when bacterial serotypes of the present study have been used in other cell models (such as professional immune cells), the expression patterns of cytokines, chemokines and TLR receptors is different from those found by us, showing a differential response pattern against one serotype over others, which would be a property of specialized cells in the recognition of structural variables in the most advanced stages of infection [26,27,28,29,30], contrary to the first phases of infection where the cells involved would develop a generic inflammatory response, without discriminating the structural variables of the pathogen, but rather acting as a first alarm signal for the assembly of a more specific response by cells with the ability to recognize virulence variability.

In addition, it should be noted that the heterogeneity in the virulence of *A. actinomycetemcomitans* is not only present among serotypes, also differences in expression induced by strains of the same serotype were observed in in vitro cell models, as strains ATCC^®^ 29524, ATCC^®^ 29522 and ATCC^®^ 43718, all belonging to serotype b, which show substantial differences between them in the expression, for example, of IL-8 and ICAM-1, in epithelial cells at 1, 3, 6, 12, and 24 h of infection, demonstrating the wide variability of virulence of this pathogen [83], and supporting the idea that the mere presence of serotype b would not necessarily implicate greater severity of periodontitis and would not be enough to establish a new pathological entity, as aggressive periodontitis was until a not long ago [87].

Finally, the study of cytokines and chemokines from all their angles is important considering the nexus among periodontal inflammation, polymicrobial biofilm and disease. Inflammation has been described with a central role in the modulation of the periodontal biofilm and in the continuous passage from a state of health, to gingivitis and later periodontitis, known as the IMPEDE model (inflammation-mediated-polymicrobial-emergence and dysbiotic-exacerbation), where the initial inflammation contributes to biofilm dysbiosis, modulating the expression of virulence factors by periodontal microorganisms (many of them known as inflammophilic bacteria) and modifying the gene expression of the host cells [88]. As such, although many bacteria are not fundamentally responsible for tissue damage, they contribute to the inflammatory environment, which is conducive to the development of other pathobionts present in the periodontal niche [89].

More studies are needed to understand the mechanisms by which *A. actinomycetemcomitans* modulates the response of different cell types, and how this host-pathogen interaction modifies the gene expression of both the microorganism and the infected cells [90].

## 5. Conclusions

*A. actinomycetemcomitans* serotypes a, b, and c have the ability to induce an increase in the expression of cytokines, chemokines, tissue destruction molecules (MMP9 and RANKL), TSLP and ICAM-1 in oral epithelial cells and macrophages, showing an increase in the earlier expression for epithelial cells compared to macrophages, and in both cases without a serotype-specific pattern associated with the increase of the production of the molecules mentioned above.

## Figures and Tables

**Table 1:** Sequences of primers used.

Target	Forward	Reverse
IL-1β	ctgtcctgcgtgttgaaaga	ttgggtaatttttgggatctaca
IL-6	gccagctatgaactccttct	gaaggcagcaggcaacac
IL-8	agatctgaagtgtgatgactc	gaagcttgtgtgctctgctgtctc
IL-18	gatagccagcctagaggtatgg	ccttgatgttatcaggaggattca
TNF-α	cagcctcttctccttcctgat	gccagagggctgattagaga
MMP-9	gccactactgtgcctttgagtc	ccctcagagaatcgccagtact
TLR-2	ctctcggtgtcggaatgtc	aggatcagcaggaacagagc
TLR-4	ccctcccctgtacccttct	tccctgccttgaataccttc
TLR-6	actgaccttcctggatgtggca	tgacctcatcttctggcagctc
TSLP	gccatgaaaactaaggctgc	cgccacaatccttgtaattg
ICAM	agcggctgacgtgtgcagtaat	tctgagacctctggcttcgtca
RANKL	tgattcatgtaggagaattaaacagg	gatgtgctgtgatccaacga
18S	ctcaacacgggaaacctcac	cgctccaccaactaagaacg

Primers were designed using the platform Ensembl Genome and Primer-BLAST (NCBH-NIH).

**Table 2:** Fold changes of mRNA relative expression in oral epithelial cells and macrophages stimulated with *A. actinomycetemcomitans.*

	Oral Epithelial Cells (OKF6/TERT2) Mean (S.D.)								Macrophage Cells (THP-1) Mean (S.D.)							
	2 h				24 h				2 h				24 h			
	a	b	c	n.i.	a	b	c	n.i.	a	b.	c.	n.i.	a	b	c	n.i.
IL-1β	2.26 (±0.1)	2.87 (±0.1)	3.48 (±0.09)	1	5.09 (±0.4)	3.65 (±0.2)	3.40 (±0.6)	1	14.32 (±0.09)	24 (±1.6)	15.52 (±1.6)	1	159.52 (±7.0)	333.16 (±2.3)	262.03 (±12.3)	1
IL-6	11.75 (±1.0)	15.39 (±0.05)	22.78 (±0.1)	1	7.91 (±0.1)	5.48 (±1.3)	7.73 (±1.0)	1	14.19 (±0.5)	13.94 (±0.6)	9.03 (±0.3)	1	124.13 (±10.7)	149 (±3.0)	164.62 (±3.4)	1
IL-8	57.69 (±1.4)	62.75 (±3.0)	96.01 (±1.6)	1	11.14 (±0.3)	3.77 (±0.1)	34.41 (±0.1)	1	15.53 (±0.9)	8.46 (±1.2)	4.40 (±0.2)	1	65.93 (±3.6)	47.60 (±5.1)	45.63 (±4.0)	1
IL-18	1.20 (±0.1)	2.11 (±0.4)	2.44 (±0.1)	1	0.86 (±0.03)	0.82 (±0.03)	0.87 (±0.05)	1	5.16 (±0.3)	3.75 (±0.2)	3.91 (±0.4)	1	35.98 (±3.2)	25.91 (±1.4)	26.85 (±1.0)	1
TNF-α	18.90 (±0.6)	20.97 (±0.5)	50.64 (±6.6)	1	2.61 (±0.2)	2.37 (±0.09)	2.11 (±0.009)	1	18.51 (±0.4)	20.99 (±0.8)	20.29 (±0.07)	1	29.54 (±0.3)	26.74 (±1.0)	26.15 (±0.7)	1
MMP-9	2.59 (±0.2)	3.31 (±0.3)	4.66 (±0.2)	1	3.64 (±0.1)	11.23 (±0.07)	35.21 (±2.3)	1	3.49 (±0.1)	6.86 (±0.4)	3.20 (±0.01)	1	4.40 (±0.2)	4.62 (±0.1)	4.3 (±0.09)	1
RANKL	2.53 (±0.2)	4.77 (±0.1)	2.99 (±0.4)	1	3.10 (±0.01)	4.17 (±0.05)	3.12 (±0.04)	1	12.10 (±0.6)	12.63 (±0.7)	12.29 (±1.5)	1	37.40 (±1.3)	32.99 (±1.3)	38.34 (±0.9)	1
TLR-2	1.66 (±0.1)	2.01 (±0.2)	1.32 (±0.1)	1	1.55 (±0.2)	1.58 (±0.01)	1.47 (±0.02)	1	3.31 (±0.1)	3.36 (±0.3)	3.41 (±0.4)	1	4.37 (±0.3)	4.55 (±0.1)	4.40 (±0.3)	1
TLR-4	2.65 (±0.1)	2.62 (±0.2)	2.85 (±0.1)	1	4.80 (±0.8)	4.75 (±1.0)	4.46 (±0.3)	1	2.28 (±0.1)	2.26 (±0.02)	2.23 (±0.2)	1	3.27 (±0.1)	3.27 (±0.2)	3.31 (±0.1)	1
TLR-6	0.97 (±0.1)	1.50 (±0.2)	1.61 (±0.09)	1	1.71 (±0.03)	1.36 (±0.01)	1.71 (±0.03)	1	2.40 (±0.3)	2.22 (±0.04)	2.14 (±0.02)	1	2.29 (±0.06)	2.77 (±0.1)	2.15 (±0.07)	1
TSLP	11.49 (±0.1)	7.38 (±0.9)	10.14 (±1.8)	1	5.57 (±0.7)	3.04 (±0.4)	8.15 (±0.4)	1	64.22 (±2.2)	58.37 (±0.5)	52.62 (±1.1)	1	66.54 (±0.8)	67.38 (±0.8)	65.44 (±1.3)	1
ICAM-1	14.29 (±0.01)	12.59 (±0.1)	16.87 (±0.7)	1	1.95 (±0.1)	2.33 (±0.2)	2.15 (±0.08)	1	12.72 (±0.8)	15.42 (±0.5)	12.42 (±0.6)	1	46.69 (±1.1)	45.48 (±1.2)	47.50 (±1.5)	1
18S	1	1	1	1	1	1	1	1	1	1	1	1	1	1	1	1

Data are represented as fold-change for 3 independent experiments, mean and standard deviation (S.D.) for each condition are showing. For relative expression, mRNA expression in non-infected (n.i.) macrophages and oral epithelial cells were considered as 1, as a reference for fold-change in expression using 18S rRNA expression levels as a normalizing endogenous control. (**a**: Sereotype a, **b**: Serotype b, **c**: Cerotype c, **n.i.**: Non-infected condition).

## References

[1] Papapanou P.N., Sanz M., Buduneli N., Dietrich T., Feres M., Fine D.H., Flemmig T.F., Garcia R., Giannobile W.V., Graziani F. (2018). Periodontitis: Consensus report of workgroup 2 of the 2017 World Workshop on the Classification of Periodontal and Peri-Implant Diseases and Conditions. J. Periodontol..

[2] Sanz M., Marco Del Castillo A., Jepsen S., Gonzalez-Juanatey J.R., D’Aiuto F., Bouchard P., Chapple I., Dietrich T., Gotsman I., Graziani F. (2020). Periodontitis and cardiovascular diseases: Consensus report. J. Clin. Periodontol..

[3] Khumaedi A.I., Purnamasari D., Wijaya I.P., Soeroso Y. (2019). The relationship of diabetes, periodontitis and cardiovascular disease. Diabetes Metab. Syndr..

[4] Polak D., Shapira L. (2018). An update on the evidence for pathogenic mechanisms that may link periodontitis and diabetes. J. Clin. Periodontol..

[5] Opacic J., Maldonado A., Ramseier C.A., Laugisch O. (2019). Einfluss der Parodontitis auf Schwangerschaft und Geburt [Influence of periodontitis on pregnancy and childbirth]. Swiss Dent. J..

[6] Dominy S.S., Lynch C., Ermini F., Benedyk M., Marczyk A., Konradi A., Nguyen M., Haditsch U., Raha D., Griffin C. (2019). *Porphyromonas gingivalis* in Alzheimer’s disease brains: Evidence for disease causation and treatment with small-molecule inhibitors. Sci. Adv..

[7] Nanayakkara S., Zhou X. (2019). Periodontitis May Be Associated With Chronic Kidney Disease, but Evidence on Causal Association Is Limited. J. Evid. Based Dent. Pract..

[8] De Oliveira Ferreira R., de Brito Silva R., Magno M.B., Carvalho Almeida A.P.C.P.S., Fagundes N.C.F., Maia L.C., Lima R.R. (2019). Does periodontitis represent a risk factor for rheumatoid arthritis? A systematic review and meta-analysis. Ther. Adv. Musculoskelet. Dis..

[9] Kalakonda B., Koppolu P., Baroudi K., Mishra A. (2016). Periodontal Systemic Connections-Novel Associations-A Review of the Evidence with Implications for Medical Practitioners. Int. J. Health Sci..

[10] Avila M., Ojcius D.M., Yilmaz O. (2009). The oral microbiota: Living with a permanent guest. DNA Cell Biol..

[11] Mosaddad S.A., Tahmasebi E., Yazdanian A., Rezvani M.B., Seifalian A., Yazdanian M., Tebyanian H. (2019). Oral microbial biofilms: An update. Eur. J. Clin. Microbiol. Infect. Dis..

[12] Hajishengallis G. (2014). Immunomicrobial pathogenesis of periodontitis: Keystones, pathobionts, and host response. Trends Immunol..

[13] Meyle J., Chapple I. (2015). Molecular aspects of the pathogenesis of periodontitis. Periodontology 2000.

[14] Rosier B.T., Marsh P.D., Mira A. (2018). Resilience of the Oral Microbiota in Health: Mechanisms that Prevent Dysbiosis. J. Dent. Res..

[15] Meuric V., Le Gall-David S., Boyer E., Acuña-Amador L., Martin B., Fong S.B., Barloy-Hubler F., Bonnaure-Mallet M. (2017). Signature of Microbial Dysbiosis in Periodontitis. Appl. Environ. Microbiol..

[16] Deng Z.L., Szafrański S.P., Jarek M., Bhuju S., Wagner-Döbler I. (2017). Dysbiosis in chronic periodontitis: Key microbial players and interactions with the human host. Sci. Rep..

[17] Slots J., Ting M. (1999). *Actinobacillus actinomycetemcomitans* and *Porphyromonas gingivalis* in human periodontal disease: Occurrence and treatment. Periodontology 2000.

[18] Hajishengallis G., Darveau R.P., Curtis M.A. (2012). The keystone-pathogen hypothesis. Nat. Rev. Microbiol..

[19] Fine D.H., Patil A.G., Velusamy S.K. (2019). *Aggregatibacter actinomycetemcomitans* (Aa) Under the Radar: Myths and Misunderstandings of Aa and Its Role in Aggressive Periodontitis. Front. Immunol..

[20] Gholizadeh P., Pormohammad A., Eslami H., Shokouhi B., Fakhrzadeh V., Kafil H.S. (2017). Oral pathogenesis of Aggregatibacter actinomycetemcomitans. Microb Pathog..

[21] Raja M., Ummer F., Dhivakar C.P. (2014). *Aggregatibacter actinomycetemcomitans*—A tooth killer?. J. Clin. Diagn. Res..

[22] Belibasakis G.N., Maula T., Bao K., Lindholm M., Bostanci N., Oscarsson J., Ihalin R., Johansson A. (2019). Virulence and Pathogenicity Properties of *Aggregatibacter actinomycetemcomitans*. Pathogens.

[23] Oscarsson J., Claesson R., Lindholm M., Höglund Åberg C., Johansson A. (2019). Tools of *Aggregatibacter actinomycetemcomitans* to Evade the Host Response. J. Clin. Med..

[24] Tuuli A., Laura K., Terhi M., Jan O., Riikka I. (2018). *Aggregatibacter actinomycetemcomitans* LPS binds human interleukin-8. J. Oral Microbiol..

[25] Takada K., Saito M., Tsuzukibashi O., Kawashima Y., Ishida S., Hirasawa M. (2010). Characterization of a new serotype g isolate of *Aggregatibacter actinomycetemcomitans*. Mol. Oral Microbiol..

[26] Melgar-Rodríguez S., Díaz-Zúñiga J., Alvarez C., Rojas L., Monasterio G., Carvajal P., Escobar A., Sanz M., Vernal R. (2016). Serotype b of *Aggregatibacter actinomycetemcomitans* increases osteoclast and memory T-lymphocyte activation. Mol. Oral Microbiol..

[27] Díaz-Zúñiga J., Monasterio G., Alvarez C., Melgar-Rodríguez S., Benítez A., Ciuchi P., García M., Arias J., Sanz M., Vernal R. (2015). Variability of the dendritic cell response triggered by different serotypes of *Aggregatibacter actinomycetemcomitans* or *Porphyromonas gingivalis* is toll-like receptor 2 (TLR2) or TLR4 dependent. J. Periodontol..

[28] Alvarez C., Benítez A., Rojas L., Pujol M., Carvajal P., Díaz-Zúñiga J., Vernal R. (2015). Differential expression of CC chemokines (CCLs) and receptors (CCRs) by human T lymphocytes in response to different *Aggregatibacter actinomycetemcomitans* serotypes. J. Appl. Oral Sci..

[29] Vernal R., Leon R., Herrera D., Garcia-Sanz J.A., Silva Sanz M. (2008). Variability in the response of human dendritic cells stimulated with Porphyromonas gingivalis or *Aggregatibacter actinomycetemcomitans*. J. Periodontal Res..

[30] Díaz-Zúñiga J., Yáñez J.P., Alvarez C., Melgar-Rodríguez S., Hernández M., Sanz M., Vernal R. (2014). Serotype-dependent response of human dendritic cells stimulated with *Aggregatibacter actinomycetemcomitans*. J. Clin. Periodontol..

[31] Henderson B., Nair S.P., Ward J.M., Wilson M. (2003). Molecular pathogenicity of the oral opportunistic pathogen *Actinobacillus actinomycetemcomitans*. Annu. Rev. Microbiol..

[32] Herbert B.A., Novince C.M., Kirkwood K.L. (2016). *Aggregatibacter actinomycetemcomitans*, a potent immunoregulator of the periodontal host defense system and alveolar bone homeostasis. Mol. Oral Microbiol..

[33] Höglund Åberg C., Haubek D., Kwamin F., Johansson A., Claesson R. (2014). Leukotoxic activity of Aggregatibacter actinomycetemcomitans and periodontal attachment loss. PLoS ONE.

[34] Groeger S.E., Meyle J. (2015). Epithelial barrier and oral bacterial infection. Periodontology 2000.

[35] Dale B.A. (2002). Periodontal epithelium: A newly recognized role in health and disease. Periodontology 2000.

[36] McCormick T.S., Weinberg A. (2010). Epithelial cell-derived antimicrobial peptides are multifunctional agents that bridge innate and adaptive immunity. Periodontology 2000.

[37] Ramage G., Lappin D.F., Millhouse E., Malcolm J., Jose A., Yang J., Bradshaw D.J., Pratten J.R., Culshaw S. (2017). The epithelial cell response to health and disease associated oral biofilm models. J. Periodontal Res..

[38] Kochi S., Yamashiro K., Hongo S., Yamamoto T., Ugawa Y., Shimoe M., Kawamura M., Hirata-Yoshihara C., Ideguchi H., Maeda H. (2017). *Aggregatibacter actinomycetemcomitans* regulates the expression of integrins and reduces cell adhesion via integrin α5 in human gingival epithelial cells. Mol. Cell. Biochem..

[39] Suga T., Mitani A., Mogi M., Kikuchi T., Fujimura T., Takeda H., Hishikawa T., Yamamoto G., Hayashi J., Ishihara Y. (2013). *Aggregatibacter actinomycetemcomitans* lipopolysaccharide stimulated epithelial cells produce interleukin-15 that regulates T cell activation. Arch. Oral Biol..

[40] Park E.K., Jung H.S., Yang H.I., Yoo M.C., Kim C., Kim K.S. (2007). Optimized THP-1 differentiation is required for the detection of responses to weak stimuli. Inflamm. Res..

[41] Daigneault M., Preston J.A., Marriott H.M., Whyte M.K., Dockrell D.H. (2010). The identification of markers of macrophage differentiation in PMA-stimulated THP-1 cells and monocyte-derived macrophages. PLoS ONE.

[42] Livak K.J., Schmittgen T.D. (2001). Analysis of relative gene expression data using real-time quantitative PCR and the 2(−Delta Delta C(T)) Method. Methods.

[43] Darveau R.P. (2010). Periodontitis: A polymicrobial disruption of host homeostasis. Nat. Rev. Microbiol..

[44] Gemmell E., Marshall R.I., Seymour G.J. (1997). Cytokines and prostaglandins in immune homeostasis and tissue destruction in periodontal disease. Periodontology 2000.

[45] Kantrong N., To T.T., Darveau R.P. (2019). Gingival Epithelial Cell Recognition of Lipopolysaccharide. Adv. Exp. Med. Biol..

[46] Yang J., Zhu Y., Duan D., Wang P., Xin Y., Bai L., Liu Y., Xu Y. (2018). Enhanced activity of macrophage M1/M2 phenotypes in periodontitis. Arch. Oral Biol..

[47] Pöllänen M.T., Salonen J.I., Uitto V.J. (2003). Structure and function of the tooth-epithelial interface in health and disease. Periodontology 2000.

[48] Graves D. (2008). Cytokines that promote periodontal tissue destruction. J. Periodontol..

[49] Pan W., Wang Q., Chen Q. (2019). The cytokine network involved in the host immune response to periodontitis. Int. J. Oral Sci..

[50] Dickinson B.C., Moffatt C.E., Hagerty D., Whitmore S.E., Brown T.A., Graves D.T., Lamont R.J. (2011). Interaction of oral bacteria with gingival epithelial cell multilayers. Mol. Oral Microbiol..

[51] Tanabe S.I., Grenier D. (2008). Macrophage tolerance response to *Aggregatibacter actinomycetemcomitans* lipopolysaccharide induces differential regulation of tumor necrosis factor-alpha, interleukin-1 beta and matrix metalloproteinase 9 secretion. J. Periodontal Res..

[52] Fine D.H., Schreiner H., Velusamy S.K. (2020). Aggregatibacter, A Low Abundance Pathobiont That Influences Biogeography, Microbial Dysbiosis, and Host Defense Capabilities in Periodontitis: The History of A Bug, And Localization of Disease. Pathogens.

[53] Nowarski R., Jackson R., Gagliani N., de Zoete M.R., Palm N.W., Bailis W., Low J.S., Harman C.C., Graham M., Elinav E. (2015). Epithelial IL-18 Equilibrium Controls Barrier Function in Colitis. Cell.

[54] Sugawara S., Uehara A., Nochi T., Yamaguchi T., Ueda H., Sugiyama A., Hanzawa K., Kumagai K., Okamura H., Takada H. (2001). Neutrophil proteinase 3-mediated induction of bioactive IL-18 secretion by human oral epithelial cells. J. Immunol..

[55] Vokurka J., Klapusová L., Pantuckova P., Kukletova M., Kukla L., Holla L.I. (2009). The association of MMP-9 and IL-18 gene promoter polymorphisms with gingivitis in adolescents. Arch. Oral Biol..

[56] Broz P., Dixit V.M. (2016). Inflammasomes: Mechanism of assembly, regulation and signalling. Nat. Rev. Immunol..

[57] Lamkanfi M., Dixit V.M. (2014). Mechanisms and functions of inflammasomes. Cell.

[58] Xue F., Shu R., Xie Y. (2015). The expression of NLRP3, NLRP1 and AIM2 in the gingival tissue of periodontitis patients: RT-PCR study and immunohistochemistry. Arch. Oral Biol..

[59] Isaza-Guzman D.M., Medina-Piedrahita V.M., Gutierrez-Henao C., Tobon-Arroyave S.I. (2017). Salivary Levels of NLRP3 Inflammasome-Related Proteins as Potential Biomarkers of Periodontal Clinical Status. J. Periodontol..

[60] Kim S., Park M.H., Song Y.R., Na H.S., Chung J. (2016). *Aggregatibacter actinomycetemcomitans*-Induced AIM2 Inflammasome Activation Is Suppressed by Xylitol in Differentiated THP-1 Macrophages. J. Periodontol..

[61] Belibasakis G.N., Johansson A. (2012). Aggregatibacter actinomycetemcomitans targets NLRP3 and NLRP6 inflammasome expression in human mononuclear leukocytes. Cytokine.

[62] Zhao P., Liu J., Pan C., Pan Y. (2014). NLRP3 inflammasome is required for apoptosis of *Aggregatibacter actinomycetemcomitans*-infected human osteoblastic MG63 cells. Acta Histochem..

[63] Mäkelä M., Salo T., Uitto V.J., Larjava H. (1994). Matrix metalloproteinases (MMP-2 and MMP-9) of the oral cavity: Cellular origin and relationship to periodontal status. J. Dent. Res..

[64] Kowolik M.J., Grant M. (1983). Myeloperoxidase activity in human gingival crevicular neutrophils. Arch. Oral Biol..

[65] Li Y.Q., Yan J.P., Xu W.L., Wang H., Xia Y.J., Wang H.J., Zhu Y.Y., Huang X.J. (2013). ADAM17 mediates MMP9 expression in lung epithelial cells. PLoS ONE.

[66] Weiler J., Mohr M., Zänker K.S., Dittmar T. (2018). Matrix metalloproteinase-9 (MMP9) is involved in the TNF-α-induced fusion of human M13SV1-Cre breast epithelial cells and human MDA-MB-435-pFDR1 cancer cells. Cell Commun. Signal..

[67] Chang M.C., Pan Y.H., Wu H.L., Lu Y.J., Liao W.C., Yeh C.Y., Lee J.J., Jeng J.H. (2019). Stimulation of MMP-9 of oral epithelial cells by areca nut extract is related to TGF-β/Smad2-dependent and -independent pathways and prevented by betel leaf extract, hydroxychavicol and melatonin. Aging.

[68] Bodet C., Chandad F., Grenier D. (2007). Inhibition of host extracellular matrix destructive enzyme production and activity by a high-molecular-weight cranberry fraction. J. Periodontal Res..

[69] Fujihara R., Usui M., Yamamoto G., Nishii K., Tsukamoto Y., Okamatsu Y., Sato T., Asou Y., Nakashima K., Yamamoto M. (2014). Tumor necrosis factor-α enhances RANKL expression in gingival epithelial cells via protein kinase A signaling. J. Periodontal Res..

[70] Usui M., Sato T., Yamamoto G., Okamatsu Y., Hanatani T., Moritani Y., Sano K., Yamamoto M., Nakashima K. (2016). Gingival epithelial cells support osteoclastogenesis by producing receptor activator of nuclear factor kappa B ligand via protein kinase A signaling. J. Periodontal Res..

[71] Zhou L., Le Y., Tian J., Yang X., Jin R., Gai X., Sun Y. (2018). Cigarette smoke-induced RANKL expression enhances MMP-9 production by alveolar macrophages. Int. J. Chron. Obstruct. Pulmon. Dis..

[72] Park S.R., Kim D.J., Han S.H., Kang M.J., Lee J.Y., Jeong Y.J., Lee S.J., Kim T.H., Ahn S.G., Yoon J.H. (2014). Diverse Toll-like receptors mediate cytokine production by Fusobacterium nucleatum and *Aggregatibacter actinomycetemcomitans* in macrophages. Infect. Immun..

[73] Rojo-Botello N.R., García-Hernández A.L., Moreno-Fierros L. (2012). Expression of toll-like receptors 2, 4 and 9 is increased in gingival tissue from patients with type 2 diabetes and chronic periodontitis. J. Periodontal Res..

[74] Lima H.R., Gelani V., Fernandes A.P., Gasparoto T.H., Torres S.A., Santos C.F., Garlet G.P., da Silva J.S., Campanelli A.P. (2010). The essential role of toll like receptor-4 in the control of *Aggregatibacter actinomycetemcomitans* infection in mice. J. Clin. Periodontol..

[75] Gelani V., Fernandes A.P., Gasparoto T.H., Garlet T.P., Cestari T.M., Lima H.R., Ramos E.S., de Souza Malaspina T.S., Santos C.F., Garlet G.P. (2009). The role of toll-like receptor 2 in the recognition of *Aggregatibacter actinomycetemcomitans*. J. Periodontol..

[76] Beklen A., Hukkanen M., Richardson R., Konttinen Y.T. (2008). Immunohistochemical localization of Toll-like receptors 1-10 in periodontitis. Oral Microbiol. Immunol..

[77] Park B.S., Song D.H., Kim H.M., Choi B.S., Lee H., Lee J.O. (2009). The structural basis of lipopolysaccharide recognition by the TLR4-MD-2 complex. Nature.

[78] Shimazu R., Akashi S., Ogata H., Nagai Y., Fukudome K., Miyake K., Kimoto M. (1999). MD-2, a molecule that confers lipopolysaccharide responsiveness on Toll-like receptor 4. J. Exp. Med..

[79] Ando-Suguimoto E.S., Benakanakere M.R., Mayer M.P.A., Kinane D.F. (2020). Distinct Signaling Pathways between Human Macrophages and Primary Gingival Epithelial Cells by *Aggregatibacter actinomycetemcomitans*. Pathogens.

[80] Takai T. (2012). TSLP expression: Cellular sources, triggers, and regulatory mechanisms. Allergol. Int..

[81] Takai T., Chen X., Xie Y., Vu A.T., Le T.A., Kinoshita H., Kawasaki J., Kamijo S., Hara M., Ushio H. (2014). TSLP expression induced via Toll-like receptor pathways in human keratinocytes. Methods Enzymol..

[82] Tsutsumi T., Nakashima K., Isoda T., Yokota M., Nishihara T. (2010). Involvement of adhesion molecule in in vitro plaque-like formation of macrophages stimulated with *Aggregatibacter actinomycetemcomitans* lipopolysaccharide. J. Periodontal Res..

[83] Shimada T., Sugano N., Nishihara R., Suzuki K., Tanaka H., Ito K. (2008). Differential effects of five *Aggregatibacter actinomycetemcomitans* strains on gingival epithelial cells. Oral Microbiol. Immunol..

[84] Shimada T., Sugano N., Ikeda K., Shimada K., Iizuka T., Ito K. (2009). Protease-activated receptor 2 mediates interleukin-8 and intercellular adhesion molecule-1 expression in response to *Aggregatibacter actinomycetemcomitans*. Oral Microbiol. Immunol..

[85] Lyck R., Enzmann G. (2015). The physiological roles of ICAM-1 and ICAM-2 in neutrophil migration into tissues. Curr. Opin. Hematol..

[86] Canonica G.W., Ciprandi G., Pesce G.P., Buscaglia S., Paolieri F., Bagnasco M. (1995). ICAM-1 on epithelial cells in allergic subjects: A hallmark of allergic inflammation. Int. Arch. Allergy Immunol..

[87] Fine D.H., Patil A.G., Loos B.G. (2018). Classification and diagnosis of aggressive periodontitis. J. Periodontol..

[88] Van Dyke T.E., Bartold P.M., Reynolds E.C. (2020). The Nexus between Periodontal Inflammation and Dysbiosis. Front. Immunol..

[89] Loos B.G., Van Dyke T.E. (2020). The role of inflammation and genetics in periodontal disease. Periodontology 2000.

[90] Umeda J.E., Longo P.L., Simionato M.R., Mayer M.P. (2013). Differential transcription of virulence genes in *Aggregatibacter actinomycetemcomitans* serotypes. J. Oral Microbiol..

